# Antibody response to Raboral VR-G® oral rabies vaccine in captive and free-ranging black-backed jackals (*Canis mesomelas*)

**DOI:** 10.4102/ojvr.v89i1.1975

**Published:** 2022-02-10

**Authors:** Katja N. Koeppel, Peter Geertsma, Brian F. Kuhn, Ockert L. van Schalkwyk, Peter N. Thompson

**Affiliations:** 1 Department of Production Animal Studies, Faculty of Veterinary Science, University of Pretoria, Onderstepoort, South Africa; 2 Centre for Veterinary Wildlife Studies, Faculty of Veterinary Science, University of Pretoria, Onderstepoort, South Africa; 3 Veterinary Services, Department of Agriculture and Rural Development, Government of South Africa, Johannesburg, South Africa; 4 Department of Geology, Faculty of Science, University of Johannesburg, Johannesburg, South Africa; 5 Office of the State Veterinarian, Department of Agriculture, Land Reform and Rural Development, Government of South Africa, Skukuza, South Africa; 6 Department of Veterinary Tropical Diseases, Faculty of Veterinary Science, University of Pretoria, Onderstepoort, South Africa; 7 Department of Migration, Max Planck Institute of Animal Behavior, Radolfzell, Germany

**Keywords:** black-backed jackal, *Canis mesomelas*, oral bait, rabies, South Africa, vaccination

## Abstract

Rabies is a zoonotic disease that remains endemic in large parts of southern Africa because of its persistence in wildlife and domestic dog vectors. The black-backed jackals (*Canis mesomelas*) is primarily the wildlife vector responsible for rabies outbreaks in northern parts of South Africa. Two trials were carried out to investigate antibody responses to the oral rabies vaccine Raboral V-RG® in black-backed jackals under captive and free-ranging conditions. In captive jackals 10/12 (83%; 95% confidence interval [CI]: 52% – 98%), seroconverted after single oral vaccination. Nine captive jackals had protective antibody titres (> 0.5 IU/mL) at 4 weeks (median: 2.1 IU/mL; inter quartile range [IQR]: 0.6–5.7) and 10 jackals had at 12 weeks (median: 3.5 IU/mL; IQR: 1.5–8.3) and three maintained antibody titres for up to 48 weeks (median: 3.4 IU/mL; IQR: 2.0–6.3). Four sites were baited with Raboral V-RG® vaccine for wild jackals, using fishmeal polymer and chicken heads. Baits were distributed by hand or from vehicle at three sites in north-eastern South Africa, with an average baiting density of 4.4 baits/km^2^ and at one site in central South Africa, at 0.12 baits/km^2^. This resulted in protective antibody titres in 3/11 jackals (27%; 95% Cl: 6–61) trapped between 3 and 12 months after baiting in north-eastern South Africa, compared with 4/7 jackals (57%; 95% Cl: 18–90) trapped after 3–18 months in central South Africa. This study shows the potential utility of oral rabies vaccination for the control of wildlife-associated rabies in north-eastern and central South Africa, but extensive studies with wider distribution of bait are needed to assess its potential impact on rabies control in wild jackals.

## Introduction

Rabies is an important zoonotic disease caused by a rhabdovirus belonging to the *Lyssavirus* genus. It is a single-stranded, negative-sense RNA virus causing fatal encephalitis in domestic animals, humans and wildlife (Swanepoel et al. 1993). In most parts of Africa, rabies is associated with domestic dogs and wildlife species have not been considered important vectors (Wandeler 1993) until more recently when it was established that jackals can sustain rabies infection without outside introductions from domestic dogs (Zulu, Sabeta & Nel 2009).

The first reported case of rabies in South Africa was in the Cape Province in 1882 from an infected dog that was brought in from England; it took two years to control the outbreak in dogs (Swanepoel et al. 1993). During that time, wildlife was apparently considered to play a minor role in rabies epidemiology until it was observed in the black-backed jackals (*Canis mesomelas*) population in 1950s in northern South Africa (Mansvelt 1956). The disease then continued to spread southwards and westwards in South Africa (Brückner, Hurter & Boshoff 1978).

The effect of rabies infection on a naïve wild carnivore population can be devastating. A 78% mortality was reported in the endangered Ethiopian wolf (*Canis simensis*) in an outbreak in the Ethiopian highlands in 2004 (Randall et al. 2004). The 2016 rabies outbreak in north-eastern South Africa resulted in 76% of stable jackal territories being lost and 93% of jackals being lost in a monitored black-backed jackals population in the Broederstroom area in north-eastern South Africa (Snyman 2020).

Poisoning of jackals to control rabies in livestock was used in the 1950s; but it was only partially effective, as disturbance to the jackal population resulted in an influx of jackals from the surrounding areas (McKenzie 1993). Rabies control by poisoning was continued until the 1980s (Bishop et al. 2010). Parenteral vaccination of cattle was started in 1977 in the endemic areas with greater effect (Brückner et al. 1978).

Oral rabies vaccines were first tested in wildlife in the field in Switzerland in 1978 (Steck et al. 1982; Wandeler 1988). Initially the Street Alabama Dufferin (SAD) strain was used, and later, over the past 30 years, 10 different vaccines were used in Europe to control rabies in red foxes (*Vulpes vulpes*) (Müller et al. 2015). Amongst these vaccines was the Raboral V-RG^®^ (Merial, United States [US], now Boehringer Ingelheim) vaccine, which was used in parts of France, Belgium and the Ukraine as part of their oral rabies vaccination strategies in wildlife between 1988 and 1993 (Müller et al. 2015).

The SAD (Berne) vaccine has been used in side-striped jackals (*Canis adustus*) and black-backed jackals. All jackals had protective antibody titres for 12 months post oral installation of vaccine and survived challenge with a field strain of the rabies virus (Bingham et al. 1995). However, the SAD (Berne) vaccine has been shown to be pathogenic in a variety of rodent species and chacma baboons (*Papio ursinus*) and is therefore not suitable for southern Africa, where chacma baboons are common (Bingham et al. 1992).

The SAG-2 (SAD Avirulent Gif) vaccine showed adequate protection in side-striped jackals, black-backed jackals and Ethiopian wolves (Sillero-Zubiri et al. 2016). Titres in side-striped jackals and black-backed jackals were protective for 12 months post-vaccination (Bingham et al. 1999). Titres > 0.5 IU/mL are recommended in dogs to be adequate to prevent rabies infection, whilst titres > 0.25 IU/mL are thought to be adequate in foxes (*Vulpes* spp.), raccoons (*Procyon lotor*) and raccoon dogs (*Nytereutes procyonoides*) (Moore et al. 2017; Office International Des Epizooties 2008). There is no single cut-off rabies antibody titre that is invariably protective as a small number of animals succumb to infection despite adequate antibody levels and some animals with no antibody titres after vaccination survive infection (Moore et al. 2017; Moore & Hanlon 2010).

The V-RG^®^ vaccine is a recombinant vaccinia virus vector vaccine expressing the rabies virus glycoprotein gene. It has successfully been used in a variety of wild carnivores such as red foxes in Europe, striped skunks (*Mephitis mephitis*), raccoons, Arctic foxes (*Vulpes lagopus*) and grey foxes (*Urocyon cinereoargenteus*) in the US and red foxes and golden jackals (*Canis aureus*) in Israel (Maki et al. 2017; Müller et al. 2015; Yakobson et al. 2006). The V-RG^®^ vaccine is 100% effective in red foxes, safe, with no risk of reverting to virulence in non-target species and stable in the environment for up to 3 weeks at environmental temperatures between 8 °C and 37 °C (Blancou et al. 1989; Maki et al. 2017).

Antibody titres and duration of immunity for the V-RG^®^ vaccine have not been established in black-backed jackals to date. The aim of this study was to investigate the duration and levels of rabies antibodies in captive black-backed jackals over a 12-month period after oral vaccination.

## Materials and methods

### Vaccine

Raboral V-RG^®^ vaccine (Boehringer Ingelheim Animal Health, US) containing 10^8^ tissue culture infective dose 50% (TCID_50_) per sachet (1.5 mL) (Maki et al. 2017) was used for the trial.

### Captive jackals

The black-backed jackals were recruited opportunistically, and juvenile jackals admitted for rehabilitation to facilities in the northern parts of South Africa during 2018 were selected for the study, with the permission of the respective rehabilitation facilities. All experimental jackals were housed in groups and were between three and six months of age at the time of vaccination. A total of 12 black-backed jackals (eight males and four females) received oral rabies vaccine. Eight jackals (six males and two females) received the vaccine in a sachet in chicken pieces and four jackals (two males and two females) received the contents of the sachet drawn up into a 1.5 mL syringe and squirted directly into the mouth. Two jackals (one male and one female) received a placebo containing a sachet of sterile water in a food item.

The jackals were sampled prior to bait administration and then at 4-, 12-, 24-, 36- and 48-weeks post-vaccination, if still in captivity. All jackals were caught with nets and hand restrained for blood collection from the cephalic vein into a 4 mL serum tube. The 12 jackals were closely monitored for the first 12 weeks after vaccination for any negative side-effects. All jackals were released back into the wild; game reserves in Mpumalanga, Limpopo and Gauteng provinces post-rehabilitation between 8 and 15 months of age.

### Free-ranging jackals

Three sites in Gauteng, north-eastern South Africa and one site on the central plateau of the Free State province (Figure 1), were selected for bait placements because of ongoing jackal research projects at these sites. It was aimed to sample 25 jackals in each study area. However, as resources were limited and capture opportunities were determined by other research activities, sampling was opportunistic and a total of 24 jackals were sampled in all the sites.

**FIGURE 1:**
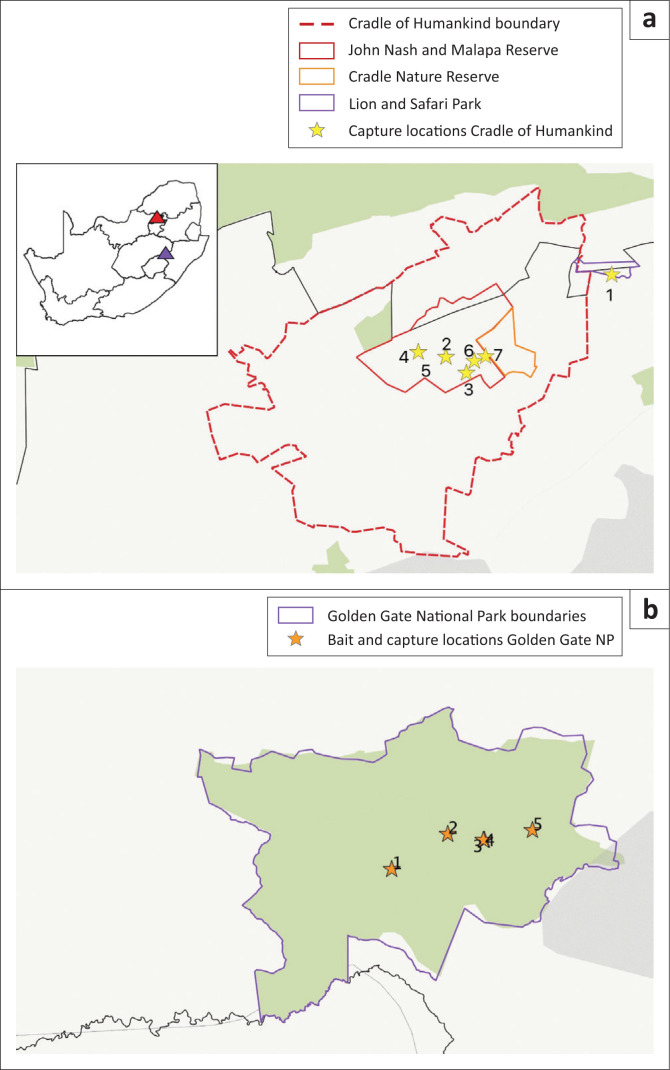
Map showing: (a) the seven capture sites at the John Nash and Malapa Reserve, Cradle Nature Reserve and the Lion and Safari Park in Gauteng and North West province (red triangle) and (b) the five baiting and capture sites at the Golden Gate National Park, Free State province (purple triangle), South Africa. Provincial and National Parks are shown in light green (the map was constructed using QGIS 3.4 using country boundaries and protected area boundaries downloaded and used with permission from protectedplanet.net and SANParks).

### Study area 1

The three sites in north-eastern South Africa were in protected areas in the Cradle of Humankind, a UNESCO World Heritage Site of 573 km^2^, within 50 km of the metropolitan areas of Johannesburg and Pretoria. The sites were: (1) the John Nash and Malapa Reserve (JNMR) (67.3 km^2^), which is situated in the centre of the Cradle, (2) the Cradle Nature Reserve (CNR) (16 km^2^), situated to the east of the JNMR, and (3) the Lion and Safari Park (LSP) (17.7 km^2^), to the north-east of the Cradle (Figure 1a). The areas are all classified as rocky highveld grassland (Mucina & Rutherford 2006).

The jackal density at JNMR and the CNR was estimated at 0.6 jackal/km^2^ from the 2016 aerial census (Hennie Visser, personal communication). The jackal density at LSP was estimated at 0.64 jackal/km^2^ on sighting and jackal call back records (Krofel et al. 2014; Snyman 2020). Six jackals were sampled in 2014 and 2015 at the LSP and all were negative for rabies antibody titres (Snyman 2020).

At the three north-eastern sites the bait was presented either in chicken heads (*n* = 270) or fishmeal polymer (*n* = 179), as these are preferred by jackals (Koeppel, Kuhn & Thompson 2020). The baits in JNMR (chicken head *n* = 206, fishmeal polymer *n* = 150) were distributed on three occasions, approximately 2–3 weeks apart between September and October 2017, whilst the CNR was baited once (chicken head: *n* = 20, fishmeal polymer: *n* = 15). The baits (chicken head: *n* = 44, fishmeal polymer: *n* = 14) at the LSP were distributed on two occasions, four weeks apart in November 2017. The baits were distributed from vehicles or on foot near jackal dens where access was limited. Baiting density varied according to road availability in the reserves. Jackals were subsequently trapped in all areas (Figure 1, seven capture sites), beginning three months after bait placement and for up to 23 months.

### Study area 2

The Golden Gate National Park (GGNP), in the Free State province of central South Africa, bordering the mountain kingdom of Lesotho, served as the fourth study site. The park covers an area of 340 km^2^ and is made up of sandstone formations interspersed with rich highveld and montane grassland and some Afromontane forests (Mucina & Rutherford 2006).

Four jackals were trapped prior to bait placement to evaluate previous rabies exposure. A total of 27 oral bait rabies vaccines were placed at five sites in the GGNP in July 2018 (Figure 1b). The annual aerial game census for 2016–2018 showed the average density of one jackal per 8.7 km^2^ or 0.12 jackal/km^2^ (Brüns A, unpublished data). The park is situated 15 km from the nearest town of Phuthadijihba with over 54 000 inhabitants (Statistics South Africa 2012).

As a result of the relative inaccessibility of the reserve, baits were placed along roads at monitored sites, with the exception of bait location 1, which was at a vulture feeding station. Bait placement was 0.1 baits/km^2^ in the GGNP. In all, 37 fishmeal polymer baits were placed at five sites in GGNP over two nights in July 2018. Jackals were trapped at three months and 18 months after baiting.

Between three months and 23 months after bait placements jackals were captured by trapping or chemical immobilisation, for blood sample collection, they were either darted from a vehicle using a X-calibre dart CO_2_ gun (Pneu-Dart INC, Williamssport, Pennsylvania, US) with a 0.5 cc dart with barbed 3/8 inch needle (Motsumi Dart, Pretoria, South Africa) or caught using a Soft catch trap Fox (Oneida Victor^®^ Animal Trap, Cleveland, Ohio, US). Traps were set at sunset and checked every 2 h throughout the night. Jackals were restrained with a net and hand injected intramuscularly with either a combination of metonil (medetomidine, Wildlife Pharmaceuticals (Pty) Ltd, Whiteriver, South Africa) 0.03 mg/kg – 0.05 mg/kg and zoletil (zolazepam and tiletamine, Pfizer Laboratories (Pty) Ltd, Johannesburg, South Africa) 3 mg/kg – 4 mg/kg or Bamanil (butorphanol 30 mg/mL, azaperone 12 mg/mL, medetomidine 12 mg/mL; Wildlife Pharmaceuticals (Pty) Ltd, Whiteriver, South Africa) 0.01 mL/kg – 0.06 mL/kg. The same drug combinations were used for darting.

Jackals were categorised by size and dentition. Juveniles were classified as less than seven months old based on their deciduous teeth (Lombaard 1971). Jackals between seven months to two years were identified as sub-adults by permanent canines and body size. Jackals greater than two years of age, according to teeth wear and body size, were classified as adults (Bingham & Purchase 2003). All jackals were ear notched for identification.

### Sample processing

Blood samples were refrigerated (4°C) shortly after collection until processing. Blood tubes were centrifuged and serum was separated and stored at –80°C prior to analysis at Agricultural Research Council-Onderstepoort Veterinary Research, South Africa. Titres were determined following the standard operating procedure of the World Organisation for Animal Health (OIE) prescribed FAVN test (Cliquet, Aubert & Sagné 1998) and were calculated against OIE dog reference serum. FAVN ≥ 0.5 IU/mL were considered to be adequate according to the World Health Organization (WHO) and OIE guidelines (Office International Des Epizooties 2008). Antibody titres > 0.2 IU/mL were considered weak positive.

### Statistical analysis

The Kruskal–Wallis rank sum test was used to compare rabies antibody titres between groups and between areas. Stata 14 (StataCorp, College Station, US) was used for statistical analysis and significance was assessed at *p* < 0.05. Maps were generated using Quantum Geographic Information System (QGIS) (version 3.4.9).

### Ethical considerations

Approval to conduct the study was obtained from the University of Pretoria Animal Ethics Committee (V149-16).

## Results

### Captive jackals

**FIGURE 2:**
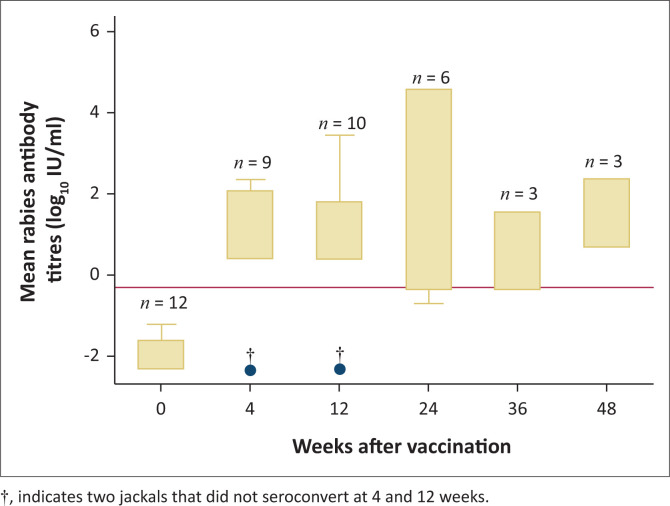
Rabies antibody titres in captive black-backed jackals (*n* = 12) given Raboral VR-G^®^ oral rabies vaccine. Red reference line = adequate antibody titre (0.5 IU/mL).

No jackals had positive FAVN (> 0.2 IU/mL) prior to vaccination (median 0.1 IU/mL, interquartile range [IQR]: 0–0.2) (Office International Des Epizooties 2008). No side-effects were observed in any of the vaccinated jackals. Of the 12 vaccinated jackals, 9 were sampled at 12 weeks, 6 at 24 weeks and 3 at 36 and 48 weeks. Of the 12 jackals, 10 that received oral rabies vaccine seroconverted at 12 weeks after oral rabies vaccination, resulting in an estimated seroconversion proportion of 83% (95% confidence interval [CI]: 0.52–0.98) (Table 1). Two jackals (jackals #22557 and #68887) did not seroconvert (Table 1). The median titre was 2.1 IU/mL (*n* = 9; IQR: 0.6–5.7) at 4 weeks post vaccination (Table 1, Figure 2). One of the jackals (#32888) could not be sampled at four weeks post vaccination.

**TABLE 1:** Rabies antibody titre (IU/mL) of 12 captive and 22 free ranging black-backed jackals in two areas (Gauteng and Free State province) of South Africa after vaccination with Raboral V-RG^®^ vaccine.

Jackal ID	Area	Bait type	Sex	Age class	Intervals after vaccination (weeks)										
					0	4	12	24	36	44	48	72	84	88	92
#22557	Captive	S	M	J	0.2	0.0	0.1	-	-	-	-	-	-	-	-
#68887	Captive	S	M	J	0.0	0.1	0.1	-	-	-	-	-	-	-	-
#24360	Captive	S	M	J	0.1	6.0†	10.5†	-	-	-	-	-	-	-	-
#32910	Captive	S	M	J	0.2	10.5†	3.5†	-	-	-	-	-	-	-	-
#32888	Captive	S	M	J	0.1	-	31.6†	-	-	-	-	-	-	-	-
#22689	Captive	S	F	J	0.0	10.5†	4.6†	-	-	-	-	-	-	-	-
Eddie	Captive	S	M	J	0.0	2.6†	3.5†	4.6†	-	-	-	-	-	-	-
#68887	Captive	S	M	J	0.1	4.6†	6.0†	1.1†	4.6†	-	2.0†	-	-	-	-
#68886	Captive	S	M	J	0.2	1.5†	1.5†	0.7†	0.7†	-	10.5†	-	-	-	-
#68889	Captive	S	F	J	0.2	1.5†	1.5†	0.5†	1.1†	-	2.0†	-	-	-	-
#65930	Captive	S	F	J	0.0	-	-	95.5†	-	-	-	-	-	-	-
#65986	Captive	S	F	J	0.0	-	-	95.5†	-	-	-	-	-	-	-
LSP1	Gauteng	FP/C	M	J	-	0.2	-	-	-	-	-	-	-	-	-
JN2	Gauteng	FP/C	F	SA	-	-	-	-	-	0.0	-	-	-	-	-
JN3	Gauteng	FP/C	F	A	-	-	-	-	-	3.5†	-	-	-	-	-
JN4	Gauteng	FP/C	F	A	-	-	-	-	-	0.0	-	-	-	-	-
JN5	Gauteng	FP/C	F	A	-	-	-	-	-	-	0.1	-	-	-	-
JN6	Gauteng	FP/C	M	A	-	-	-	-	-	-	-	-	0.4†	-	-
CR3	Gauteng	FP/C	F	A	-	-	-	-	-	-	-	-	-	0.4†	-
JN7	Gauteng	FP/C	F	A	-	-	-	-	-	-	-	-	-	0.1	-
JN8	Gauteng	FP/C	F	A	-	-	-	-	-	-	-	-	-	0.0	-
JN13	Gauteng	FP/C	M	A	-	-	-	-	-	-	-	-	-	-	0.1
JN14	Gauteng	FP/C	M	SA	-	-	-	-	-	-	-	-	-	-	0.0
GG1	Free State	Co	M	A	0.1	-	-	-	-	-	-	-	-	-	-
GG2	Free State	Co	F	SA	0.1	-	-	-	-	-	-	-	-	-	-
GG3	Free State	Co	M	SA	0.1	-	-	-	-	-	-	-	-	-	-
GG4	Free State	Co	F	SA	0.1	-	-	-	-	-	-	-	-	-	-
GG5	Free State	FP	M	SA	-	-	0.9†	-	-	-	-	-	-	-	-
GG6	Free State	FP	F	A	-	-	0.1	-	-	-	-	-	-	-	-
GG7	Free State	FP	F	SA	-	-	0.1	-	-	-	-	-	-	-	-
GG8	Free State	FP	F	A	-	-	1.5†	-	-	-	-	-	-	-	-
GG11	Free State	FP	F	J	-	-	-	-	-	-	-	0.4†	-	-	-
GG12	Free State	FP	F	J	-	-	-	-	-	-	-	0.2	-	-	-
GG13	Free State	FP	F	J	-	-	-	-	-	-	-	1.1†	-	-	-

Bait type: Co, control; S, V-RG sachet; FP, fish polymer with V-RG; C, chicken head with V-RG.

Age Classification: J, juvenile; SA, subadult; A, adult.

† , Seropositive titres: > 0.2 IU/mL (Office International Des Epizooties, 2008).

The median titre at 12 weeks was 3.5 IU/mL (*n* = 10; IQR: 1.5–8.3). At 24 weeks the median was 2.9 IU/mL (*n* = 6; IQR: 0.8–72.8), at 36 weeks the median was 1.1 IU/mL (*n* = 3; IQR: 0.9–2.9), and at 48 weeks the median was 3.4 IU/mL (*n* = 3; IQR: 2–6.3) (Figure 1). The two jackals which received placebo vaccine did not seroconvert (data not shown). Antibody titres were significantly higher at 4-, 12- and 24- weeks (*p* = 0.037) in jackals when the vaccine was administered directly into the mouth (*n* = 4) compared with presentation of vaccine in food items (*n* = 8) (Figure 1-A1).

### Free-ranging jackals

In the north-eastern part of South Africa (Gauteng reserves, Study area 1), baiting density varied; the JNMR was baited with 5.3 baits/km^2^, CNR received 2.2 baits/km^2^ and the LSP was baited with 3.3 baits/km^2^ (Table 2). The average baiting density for these reserves was 4.4 baits/km.^2^

**TABLE 2:** Jackal population per square kilometre, bait density, number of jackals sampled after vaccination (*n*) and number of rabies antibody (Ab) positive jackals and percentage of positive jackals in three Gauteng and one Free State Reserves from 2018 to 2020, South Africa.

Reserve	Jackal/km^2^	Baits/km^2^	Jackals tested (*n*)	Ab Positive jackals	% positive
John Nash and Malapa Reserve (GP)	0.6	5.3	9	2	22
Cradle Nature Reserve (GP)	0.6	2.2	1	1	100
Lion and Safari Park (GP)	0.64	3.3	1	0	0
Golden Gate National Park (FS)	0.12	0.1	7	4	57

Ab, antibody; GP, Gauteng; FS, Free State.

A total of 11 jackals (4 males, 7 females) were caught between 3 and 23 months after baiting at the three north-eastern sites. Of the 11 jackals (27%; 95% CI: 6–61), 3 had antibody titres against rabies virus (Table 2). One jackal had adequate levels (3.5 IU/mL) at 44 weeks after vaccination and two jackals had low rabies antibody titres at 84 (0.4 IU/mL) and 88 (0.4 IU/mL) weeks (Table 1).

At GGNP (Study area 2) four jackals (two males, two females; ~10% of population) were caught prior to bait placement; none had detectable rabies antibody titres (Table 1 GG1–GG4). Baits were placed at five sites. No baits were taken at Sites 5 and 2 as there was no jackal activity in those areas. All baits were taken at the Sites 1 and 3, and 40% of bait was consumed at Site 4 (Figure 1b).

After the placement of rabies bait at GGNP for 3 and 18 months, four and three jackals were captured, respectively. No jackal was captured more than once, as individuals were identified by ear notching. Of the seven jackals (one male, six females) captured after baiting, four (57%; 95% CI: 18–90) had detectable rabies antibody titres (> 0.2 IU/mL), three of which (42%; 95% CI: 10–80) were ≥ 0.5 IU/mL (Table 1). The three jackals captured at 18 months post vaccination were juveniles estimated at below 14 weeks of age. There was no significant difference in titres between the jackals from the two provinces (*p* = 0.108).

## Discussion

This study has shown that the Raboral V-RG^®^ oral rabies vaccine results in adequate antibody titres for up to 12 months after vaccination in captive black-backed jackals. The titres in the captive jackals indicated that protective immunity was likely from 4 to 48 weeks after initial vaccination without a booster vaccination, similar to foxes vaccinated with recombinant vaccine, which lasted for 48 weeks (Brochier et al. 1988) and raccoons with detectable titres, which lasted for up to 56 weeks (Brown et al. 2011). The FAVN was not validated for black-backed jackals in this study but has been validated in the closely related golden jackals (Yakobson et al. 2006) and we assumed there is no species difference.

Only a single bait was given in this study, as recommended by the manufacturer as oral booster vaccination with V-RG^®^ within one month of initial vaccination did not improve antibody titres in foxes (Lambot et al. 2001). This is in contrast to wild dogs (*Lycaon pictus*) in which neutralising antibodies are short-lived without a booster vaccination with both oral and parenteral vaccines (Hofmeyr et al. 2000). Golden jackals showed adequate antibody response to the V-RG^®^ vaccine with a seroconversion of 44% in a controlled environment and 78% of vaccinated jackals surviving challenge with Rabies Virus (RABV), with a vaccine efficacy of 83% (Yakobson et al. 1999).

Two jackals in the captive trial in this study did not seroconvert at either 4 or 12 weeks. This is similar to the study in wild dogs were two of eleven dogs did not seroconvert at four weeks (Knobel, Liebenberg & Du Toit 2003). It is likely that the two jackals swallowed the whole food item without penetrating the sachet and the vaccine would have been rapidly degraded in the gastrointestinal tract (Vos et al. 2017). Mean titres were significantly higher in jackals that received the vaccine squirted directly into mouth versus in food items. This is most likely because of more effective exposure of vaccine to the mucous membranes when squirted directly into the mouth (Bingham et al. 1995; Grosenbaugh et al. 2007; Wandeler 1988).

In a combined sample size of 10 jackals, all tested negative prior to bait placement in the two areas of South Africa; however, the sample size was too small to rule out any antibody levels in the population. Presence of antibodies in a population has been linked to the sampling period with antibodies being more likely present during rabies epidemics (Bingham & Foggin 1993) and also the fact that jackals can survive natural rabies infection, although this is very rare (Bellan et al. 2012).

After oral bait rabies vaccination, 27% of the jackals trapped at study area 1 had detectable rabies antibody titres, resulting from an average baiting density of 4.4 baits/km^2^. This was similar to the 29.5% seropositivity reported in golden jackals and red foxes in Israel resulting from a baiting density of 14–19 baits/km^2^ (Yakobson et al. 2006). It is possible that some of the titres were because of previous exposure; however, there were no recorded outbreaks in the area from 2017 to 2019 making it unlikely to be associated with exposure to wild RABV (Koeppel, Van Schalkwyk & Thompson 2021). The percentage of jackals having antibody titres at GGNP was higher, with 57% of jackals captured having detectable titres. Higher baiting density might improve seroconversion in north-eastern South Africa but continuous baiting is essential to ensure on-going protection as there is a large turnover in jackal population over time.

Bait density was low in both areas, but bait placement by hand and from vehicles makes it possible to target more suitable habitat and den sites, making it more efficient than distribution by aircraft. In raccoons, hand baiting was more effective than helicopter distribution, especially in urban areas (Boulanger et al. 2008).

In the north-eastern parts, jackals were captured for up to 92 weeks after placement of rabies baits, which might have resulted in lower or no titres as antibody titres decrease with time; in comparison, in the central parts jackals were only tested up to 72 weeks after bait placement. No tetracycline biomarkers were tested in the jackals in this study, which would have shown previous exposure despite no antibody titre, as only subsequently released live animals were tested. The removal of canine teeth is to date the best method for detecting tetracycline in teeth in meso-predator (Robardet et al. 2012). Removal of teeth in a free-ranging animal might affect its hunting ability and survival and was therefore not performed (Verstraete et al. 1996).

There was an estimated population of 0.60–0.64 jackal per km^2^ at the study area 1 (Snyman 2020). This is in contrast to area 2 where there was 0.12 jackal per km^2^, only one sixth of the population density of the north-eastern sites. There was also a difference in bait type used with both chicken heads and fishmeal polymer used in north-eastern sites and only fishmeal in central area. It is unlikely that the bait type used had a great effect on uptake at the central area as there is low density of non-target species in the area and therefore competition for bait is reduced and jackals have been shown to consume both baits equally (Koeppel et al. 2020).

The higher percentage of seropositive jackals at the central site might be associated with a reduced population turnover, but further studies are needed to verify this. Higher population turnover will require higher density of bait placement or more frequent application to result in adequate antibody levels in the population (Holmala & Kauhala 2006). It has been shown that rabies antibody titres can be transferred from vaccinated foxes to kits; the rabies antibody titre in the fox kits declined between 45 days and 75 days after birth to zero (Blasco et al. 2001).

Two out of three juvenile jackals at the central site had rabies antibody titres present in our study, one above 0.5 IU/mL. They were all below 14 weeks of age according to their dentition (Lombaard 1971) and were captured 72 weeks after bait placement; therefore, they could not have been exposed to the original bait but might have received bait-induced antibodies indirectly via placental transfer and colostrum (Chappuis 1998).

Rabies in jackals poses a risk not only to humans, livestock and domestic animals, but also to endangered wildlife species. Rabid jackals resulted in two rabies outbreaks, which caused up to 87% mortality in affected wild dog packs at the Madikwe Game Reserve in the northwest of South Africa (Hofmeyr et al. 2000, 2004).

In foxes it has been suggested that the presence of 60% immune animals will prevent the spread of rabies, whilst strategic placement of vaccine along natural barriers can be more efficient, requiring fewer animals to be vaccinated (Haydon et al. 2006; Wandeler et al. 1988). This could be applied to areas such as the GGNP (central area) were jackals travel nearly exclusively through the valleys. Bait placement in this study was along the game paths and roads frequented by jackals, only covering a small area of the park, yet a high estimated proportion of jackals (57%) had titres after bait placement, supporting the notion that natural barriers such as mountain ranges or rivers can aid in the distribution of baits.

As a result of the low numbers of wild jackals included in this study, there is a high degree of uncertainty regarding the initial seroprevalence of rabies antibody titres in the study areas and the seroprevalence post-vaccination. Antibodies detected at ≤ 0.2 IU/mL could be related to non-antibody neutralising factors and therefore were considered negative in this study (Gold et al. 2020).

Density of jackals was estimated based on aerial counts, sightings and call backs, which could have underestimated population numbers. Furthermore, the inability to follow up on individual animals also did not allow us to conclusively demonstrate seroconversion. However, the study in captive jackals showed that oral vaccination can result in adequate levels of long-lived antibody titres in vaccinated animals. More intensive studies over larger areas could yield more precise estimates of the level of baiting required in free-ranging jackal populations to ensure adequate population immunity to rabies virus.

The optimal bait density might vary between regions and density of jackal populations. Ideally jackal carcasses in areas covered by vaccination should be evaluated for tetracycline biomarker presence (Robardet et al. 2012) and antibody titres (fresh carcass only) to determine the effect of bait placement in the areas and more precise estimates of seroprevalence and seroconversion rate should be correlated with bait density and land use type.

## Conclusion

Captive black-backed jackals vaccinated with Raboral V-RG^®^ vaccine in oral bait resulted in adequate antibody titres against rabies for a period of up to 48 weeks in the majority of animals. Use of the Raboral V-RG^®^ vaccine in free-ranging jackals in four protected areas resulted in rabies antibody titres detected in-between 27% and 57% of the sampled population supporting the use of oral rabies vaccination for the control of wildlife-associated rabies. The response to vaccination was likely to be influenced by, amongst other factors, baiting density, jackal density and population turnover. More research is required to evaluate the impact of oral rabies vaccination on population immunity and the occurrence of rabies outbreaks in jackals.

## Figures and Tables

**FIGURE 1-A1:**
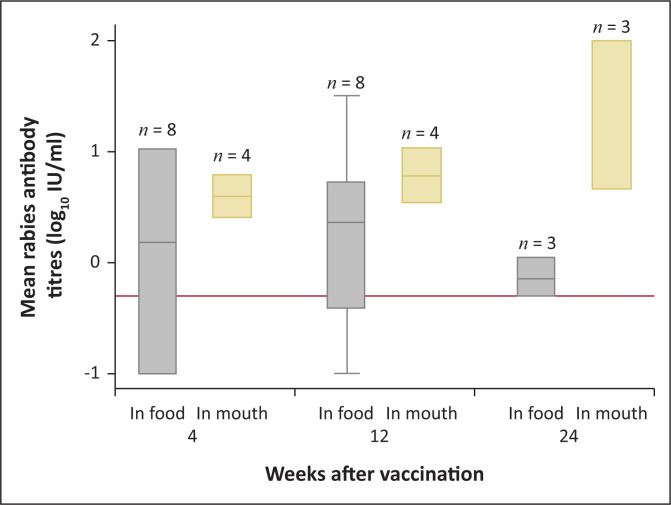
Comparison of rabies antibody titres at 4, 12 and 24 weeks after administration of Raboral VR-G^®^ rabies vaccine in food versus orally in captive black-backed jackals. Red reference line = adequate antibody titre (0.5 IU/mL).

## References

[CIT0001] Bellan, S.E., Cizauskas, C.A., Miyen, J., Ebersohn, K., Küsters, M., Prager, K.C. et al., 2012, ‘Black-backed jackal exposure to rabies virus, canine distemper virus, and Bacillus anthracis in Etosha National Park, Namibia’, *Journal of Wildlife Diseases* 48(2), 371–381. 10.7589/0090-3558-48.2.371 22493112 PMC5479413

[CIT0002] Bingham, J. & Foggin, C.M., 1993, ‘Jackal rabies in Zimbabwe’, *The Onderstepoort Journal of Veterinary Research* 60, 365–366. 7777321

[CIT0003] Bingham, J., Foggin, C.M., Gerber, H., Hill, F.W.G., Kappeler, A., King, A.A. et al., 1992, ‘Pathogenicity of SAD rabies vaccine given orally in chacma baboons (Papio ursinus)’, *Veterinary Record* 131, 55–56. 10.1136/vr.131.3.55 1441164

[CIT0004] Bingham, J., Kappeler, A., Hill, F.W.G., King, A.A., Perry, B.D. & Foggin, C.M., 1995, ‘Efficacy of sad (Berne) rabies vaccine given by the oral route in 2 species of Jackal (Canis-Mesomelas and Canis-Adustus)’, *Journal of Wildlife Diseases* 31(3), 416–419. 10.7589/0090-3558-31.3.416 8592368

[CIT0005] Bingham, J. & Purchase, G.K., 2003, ‘Age determination in jackals (*Canis adustus* Sundevall, 1846, and *Canis mesomelas* Schreber, 1778; Carnivora: Canidae) with reference to the age structure and breeding patterns of jackal populations in Zimbabwe’, *African Zoology* 38(1), 153–160. 10.1080/15627020.2003.11657203

[CIT0006] Bingham, J., Schumacher, C.L., Hill, F.W.G. & Aubert, A., 1999, ‘Efficacy of SAG-2 oral rabies vaccine in two species of jackal (*Canis adustus* and *Canis mesomelas*)’, *Vaccine* 17(6), 551–558. 10.1016/S0264-410X(98)00233-3 10075161

[CIT0007] Bishop, G.C., Durrheim, D.N., Kloeck, P.E., Godlonton, J.D., Bingham, J., Speare, R. et al., 2010, *Rabies: Guide for the medical, veterinary and allied professions*, in L. Blumberg et al. (eds.), 2nd edn., Goverment Printers, Pretoria, viewed 03 January 2019, from https://www.daff.gov.za/vetweb/Pamphlets&Information/Rabies/Rabies_Guide_2010_small.pdf.

[CIT0008] Blancou, J., Artois, M., Brochier, B., Thomas, I., Pastoret, P.P., Desmettre, P. et al., 1989, ‘Safety and efficacy of an antirabies vaccine consisting of recombinant vaccinia-rabies virus administered orally to the fox, dog and cat’, *Annales de Recherches Vétérinaires* 20, 195–204. 2751231

[CIT0009] Blasco, E., Lambot, M., Barrat, J., Cliquet, F., Brochier, B., Renders, C. et al., 2001, ‘Kinetics of humoral immune response after rabies VR-G oral vaccination of captive fox cubs (*Vulpes vulpes*) with or without maternally derived antibodies against the vaccine’, *Vaccine* 19(32), 4805–4815. 10.1016/S0264-410X(01)00211-0 11535333

[CIT0010] Boulanger, J.R., Bigler, L.L., Curtis, P.D., Lein, D.H. & Lembo, A.J., 2008, ‘Comparison of suburban vaccine distribution strategies to control raccoon rabies’, *Journal of Wildlife Diseases* 44(4), 1014–1023. 10.7589/0090-3558-44.4.1014 18957661

[CIT0011] Brochier, B.M., Languet, B., Blancou, J., Kieny, M.P., Lecocq, J.P., Costy, F. et al., 1988, ‘Use of recombinant vaccinia-rabies virus for oral vaccination of fox cubs (*Vulpes vulpes*, L) against rabies’, *Veterinary Microbiology* 18(2), 103–108. 10.1016/0378-1135(88)90055-7 3218072

[CIT0012] Brown, L.J., Rosatte, R.C., Fehlner-Gardiner, C., Knowles, M.K., Bachmann, P., Davies, J.C. et al., 2011, ‘Immunogenicity and efficacy of two rabies vaccines in wild-caught, captive raccoons’, *Journal of Wildlife Diseases* 47(1), 182–194. 10.7589/0090-3558-47.1.182 21270007

[CIT0013] Brückner, G.K., Hurter, L.R. & Boshoff, J.N., 1978, ‘Field observations on the occurrence of rabies in cattle in the magisterial districts of Soutpansberg and Messina’, *Journal of the South African Veterinary Association* 49, 33–36. 702508

[CIT0014] Chappuis, G., 1998, ‘Neonatal immunity and immunization in early age: Lessons from veterinary medicine’, *Vaccine* 16(14–15), 1468–1472. 10.1016/S0264-410X(98)00110-8 9711790 PMC7130764

[CIT0015] Cliquet, F., Aubert, M. & Sagné, L., 1998, ‘Development of a fluorescent antibody virus neutralisation test (FAVN test) for the quantitation of rabies-neutralising antibody’, *Journal of Immunological Methods* 212(1), 79–87. 10.1016/S0022-1759(97)00212-3 9671155

[CIT0016] Gold, S., Donnelly, C.A., Nouvellet, P. & Woodroffe, R., 2020, ‘Rabies virus-neutralising antibodies in healthy, unvaccinated individuals: What do they mean for rabies epidemiology?’, *PLoS Neglected Tropical Diseases* 14(2), e0007933. 10.1371/journal.pntd.0007933 32053628 PMC7017994

[CIT0017] Grosenbaugh, D.A., Maki, J.L., Rupprecht, C.E. & Wall, D.K., 2007, ‘Rabies challenge of captive striped skunks (*Mephitis mephitis*) following oral administration of a live vaccinia-vectored rabies vaccine’, *Journal of Wildlife Diseases* 43(1), 124–128. 10.7589/0090-3558-43.1.124 17347402

[CIT0018] Haydon, D.T., Randall, D.A., Matthews, L., Knobel, D.L., Tallents, L.A., Gravenor, M.B. et al., 2006, ‘Low-coverage vaccination strategies for the conservation of endangered species’, *Nature* 443, 692–695. 10.1038/nature05177 17036003

[CIT0019] Hofmeyr, M., Bingham, J., Lane, E.P., Ide, A. & Nel, L., 2000, ‘Rabies in African wild dogs (*Lycaon pictus*) in the Madikwe Game Reserve, South Africa’, *Veterinary Record* 146(2), 50–52. 10.1136/vr.146.2.50 10678814

[CIT0020] Hofmeyr, M., Hofmeyr, D., Nel, L. & Bingham, J., 2004, ‘A second outbreak of rabies in African wild dogs (*Lycaon pictus*) in Madikwe Game Reserve, South Africa, demonstrating the efficacy of vaccination against natural rabies challenge’, *Animal Conservation* 7(2), 193–198. 10.1017/S1367943004001234

[CIT0021] Holmala, K. & Kauhala, K., 2006, ‘Ecology of wildlife rabies in Europe’, *Mammal Review* 36(1), 17–36. 10.1111/j.1365-2907.2006.00078.x

[CIT0022] Knobel, D.L., Liebenberg, A. & Du Toit, J.T., 2003, ‘Seroconversion in captive African wild dogs (*Lycaon pictus*) following administration of a chicken head bait/SAG-2 oral rabies vaccine combination’, *Onderstepoort Journal of Veterinary Research* 70, 73–77. 12825684

[CIT0023] Koeppel, K.N., Kuhn, B.F. & Thompson, P.N., 2020, ‘Oral bait preferences for rabies vaccination in free-ranging black-backed jackal (*Canis mesomelas*) and non-target species in a multi-site field study in a peri-urban protected area in South Africa’, *Preventive Veterinary Medicine* 175, 104867. 10.1016/j.prevetmed.2019.104867 31927421

[CIT0024] Koeppel, K.N., Van Schalkwyk, O.L. & Thompson, P.N., 2021, ‘Patterns of rabies cases in South Africa between 1993-2019, including the role of wildlife’, *Transboundary and Emerging Diseases* 69(2), 1–13. 10.1111/tbed.14080 33738979

[CIT0025] Krofel, M., Melzheimer, J., Portas, R. & Žagar, A., 2014, ‘Use of acoustic method to survey black-backed jackal (*Canis mesomelas*)’, in *First International Jackal Symposium*, pp. 33–37, Veliko Gradiste, Serbia.

[CIT0026] Lambot, M., Blasco, E., Barrat, J. & Cliquet, F., 2001, ‘Humoral and cell-mediated immune responses of foxes (*Vulpes vulpes*) after experimental primary and secondary oral vaccination using SAG 2 and V-RG vaccines’, *Vaccine* 19(13–14), 1827–1835. 10.1016/S0264-410X(00)00321-2 11166908

[CIT0027] Lombaard, L.J., 1971, ‘Age determination and growth curves in the black-backed jackal, *Canis mesomelas*’, *Annals of the Transvaal Museum* 27, 135–175.

[CIT0028] Maki, J., Guiot, A.L., Aubert, M., Brochier, B., Cliquet, F., Hanlon, C.A. et al., 2017, ‘Oral vaccination of wildlife using a vaccinia – Rabies – Glycoprotein recombinant virus vaccine (RABORAL VRG^®^): A global review’, *Veterinary Research* 48, 1–26. 10.1186/s13567-017-0459-9 28938920 PMC5610451

[CIT0029] Mansvelt, P.R., 1956, ‘Rabies in the Northern Transvaal (1950 outbreak)’, *Journal of the South African Veterinary Medical Association* 27, 167–178.

[CIT0030] McKenzie, A.A., 1993, ‘Biology of the black-backed jackal Canis mesomelas with reference to rabies’, *The Onderstepoort Journal of Veterinary Research* 60, 367–371. 7777322

[CIT0031] Moore, S.M., Gilbert, A., Vos, A., Freuling, C.M., Ellis, C., Kliemt, J. et al., 2017, ‘Rabies virus antibodies from oral vaccination as a correlate of protection against lethal infection in wildlife’, *Tropical Medicine and Infectious Disease* 2(3), 31. 10.3390/tropicalmed2030031 PMC6082110 30270888

[CIT0032] Moore, S.M. & Hanlon, C.A., 2010, ‘Rabies-specific antibodies: Measuring surrogates of protection against a fatal disease’, *PLoS Neglected Tropical Diseases* 4(3), e595. 10.1371/journal.pntd.0000595 20231877 PMC2834733

[CIT0033] Mucina, L. & Rutherford, M.C., 2006, *The vegetation of South Africa, Lesotho and Swaziland*, South African Biodiversity Institute, Pretoria.

[CIT0034] Müller, T.F., Schröder, R., Wysocki, P., Mettenleiter, T.C. & Freuling, C.M., 2015, ‘Spatio-temporal use of oral rabies vaccines in fox rabies elimination programmes in Europe’, *PLoS Neglected Tropical Diseases* 9(8), e0003953. 10.1371/journal.pntd.0003953 26280895 PMC4539187

[CIT0035] Office International Des Epizooties, 2008, *Rabies*, viewed 12 November 2018, from http://www.oie.int/fileadmin/Home/eng/Health_standards/tahm/2.01.13_RABIES.pdf.

[CIT0036] Randall, D.A., Williams, S.D., Kuzmin, I.V., Rupprecht, C.E., Tallents, L.A., Tefera, Z. et al., 2004, ‘Rabies in endangered Ethiopian wolves’, *Emerging Infectious Diseases* 10(12), 2214–2217. 10.3201/eid1012.040080 15663865 PMC3323365

[CIT0037] Robardet, E., Demerson, J.M., Andrieu, S. & Cliquet, F., 2012, ‘First European interlaboratory comparison of tetracycline and age determination with red fox teeth following oral rabies vaccination programs’, *Journal of Wildlife Diseases* 48(4), 858–868. 10.7589/2011-07-205 23060487

[CIT0038] Sillero-Zubiri, C., Marino, J., Gordon, C.H., Bedin, E., Hussein, A., Regassa, F. et al., 2016, ‘Feasibility and efficacy of oral rabies vaccine SAG2 in endangered Ethiopian wolves’, *Vaccine* 34(40), 4792–4798. 10.1016/j.vaccine.2016.08.021 27543453

[CIT0039] Snyman, M., 2020, *Population dynamics of Canis mesomelas in the peri-urban area, Broederstroom, South Africa*, Tswane University of Technology.

[CIT0040] Statistics South Africa, 2012, *Census 2011*, viewed 01 June 2020, from http://www.statssa.gov.za.

[CIT0041] Steck, F., Wandeler, A., Bichsel, P., Capt, S., Haeflinger, U. & Schneider, L., 1982, ‘Oral immunization of foxes against rabies Laboratory and field studies’, *Comparative Immunology, Microbiology and Infectious Diseases* 5(1–3), 165–171. 10.1016/0147-9571(82)90031-5 6751678

[CIT0042] Swanepoel, R., Barnard, B.J., Meredith, C.D., Bishop, G.C., Brückner, G.K., Foggin, C.M. et al., 1993, ‘Rabies in southern Africa’, *The Onderstepoort Journal of Veterinary Research* 60, 325–346. 7777317

[CIT0043] Verstraete, F.J.M., Aarde, R.J. Van, N, B.A., Mauer, E. & Kassw, P.H., 1996, ‘The dental pathology of feral cats on Marion Island, Part I: Congenital, developmental and traumatic abnormalities’, *Journal of Comparative Pathology* 115(3), 265–282. 10.1016/S0021-9975(96)80084-3 8923237

[CIT0044] Vos, A., Freuling, C.M., Hundt, B., Kaiser, C., Nemitz, S., Neubert, A. et al., 2017, ‘Oral vaccination of wildlife against rabies: Differences among host species in vaccine uptake efficiency’, *Vaccine* 35(32), 3938–3944. 10.1016/j.vaccine.2017.06.022 28641888

[CIT0045] Wandeler, A.I., 1988, ‘Control of wildlife rabies: Europe’, in J.B. Campbell & K.M. Charlton (eds.), *Rabies: Developments in veterinary virology*, vol. 7, pp. 365–380, Springer, Boston, MA.

[CIT0046] Wandeler, A.I., 1993, ‘Wildlife rabies in perspective’, *The Onderstepoort Journal of Veterinary Research* 60, 347–350. 7777318

[CIT0047] Wandeler, A.I., Capt, S., Kappeler, A. & Hauser, R., 1988, ‘Oral immunization of wildlife against rabies: Concept and first field experiments’, *Reviews of Infectious Diseases* 10(suppl. 4), S649–S653. 10.1093/clinids/10.Supplement_4.S649 3206075

[CIT0048] Yakobson, B.A., King, R.J., David, D., Sheichat, N., Rotenderg, D., Dveres, N., et al., 1999, ‘Comparative efficacy of two oral vaccines in captive jackals (Canis aureus)’, in Rabies in the Americas meeting, November 14–19, California, US.

[CIT0049] Yakobson, B.A., King, R., Amir, S., Devers, N., Sheichat, N., Rutenberg, D. et al., 2006, ‘Rabies vaccination programme for red foxes (*Vulpes vulpes*) and golden jackals (*Canis aureus*) in Israel (1999–2004)’, *Developments in Biologicals* 125, 133–140. 16878470

[CIT0050] Zulu, G.C., Sabeta, C.T. & Nel, L.H., 2009, ‘Molecular epidemiology of rabies: Focus on domestic dogs (*Canis familiaris*) and black-backed jackals (*Canis mesomelas*) from northern South Africa’, *Virus Research* 140(1–2), 71–78. 10.1016/j.virusres.2008.11.004 19061924

